# Human pleural fluid triggers global changes in the transcriptional landscape of *Acinetobacter baumannii* as an adaptive response to stress

**DOI:** 10.1038/s41598-019-53847-2

**Published:** 2019-11-21

**Authors:** Jasmine Martinez, Jennifer S. Fernandez, Christine Liu, Amparo Hoard, Anthony Mendoza, Jun Nakanouchi, Nyah Rodman, Robert Courville, Marisel R. Tuttobene, Carolina Lopez, Lisandro J. Gonzalez, Parvin Shahrestani, Krisztina M. Papp-Wallace, Alejandro J. Vila, Marcelo E. Tolmasky, Robert A. Bonomo, Rodrigo Sieira, Maria Soledad Ramirez

**Affiliations:** 1 Center for Applied Biotechnology Studies, Department of Biological Science, College of Natural Sciences and Mathematics, California State University Fullerton, Fullerton, California USA; 2 Instituto de Biología Molecular y Celular de Rosario (IBR, CONICET-UNR), Rosario, Argentina; 3 Medical Service and GRECC, Louis Stokes Cleveland Department of Veterans Affairs Medical Center, Cleveland, Ohio USA; 4 Departments of Medicine, Pharmacology, Molecular Biology and Microbiology, Biochemistry, Proteomics and Bioinformatics, Case Western Reserve University School of Medicine, Cleveland, Ohio USA; 5 CWRU-Cleveland VAMC Center for Antimicrobial Resistance and Epidemiology (Case VA CARES), Cleveland, Ohio USA; 6 Fundación Instituto Leloir – IIBBA CONICET, Buenos Aires, Argentina

**Keywords:** Pathogens, Infection

## Abstract

*Acinetobacter baumannii* is a feared, drug-resistant pathogen, characterized by its ability to resist extreme environmental and nutrient-deprived conditions. Previously, we showed that human serum albumin (HSA) can increase foreign DNA acquisition specifically and alter the expression of genes associated with pathogenicity. Moreover, in a recent genome-wide transcriptomic study, we observed that pleural fluid (PF), an HSA-containing fluid, increases DNA acquisition, can modulate cytotoxicity, and control immune responses by eliciting changes in the *A*. *baumannii* metabolic profile. In the present work, using more stringent criteria and focusing on the analysis of genes related to pathogenicity and response to stress, we analyzed our previous RNA-seq data and performed phenotypic assays to further explore the impact of PF on *A*. *baumannii*’s microbial behavior and the strategies used to overcome environmental stress. We observed that PF triggered differential expression of genes associated with motility, efflux pumps, antimicrobial resistance, biofilm formation, two-component systems (TCSs), capsule synthesis, osmotic stress, and DNA-damage response, among other categories. Phenotypic assays of *A*. *baumannii* A118 and two other clinical *A*. *baumannii* strains, revealed differences in their responses to PF in motility, biofilm formation, antibiotic susceptibility, osmotic stress, and outer membrane vesicle (OMV) production, suggesting that these changes are strain specific. We conclude that *A*. *baumannii’s* pathoadaptive responses is induced by HSA-containing fluids and must be part of this bacterium armamentarium to persist in hostile environments.

## Introduction

*Acinetobacter baumannii* is a multi-drug-resistant (MDR) pathogen accounting for high mortality rates in immunocompromised patients. Capable of persisting under desiccation, nutrient starvation, and exposure to high concentrations of antimicrobial agents, *A*. *baumannii* has a marked advantage to prevail in clinical settings^1–4^. So alarming is the threat of *A*. *baumannii* that it was ranked as a “priority one” pathogen for antibiotic research and development by the World Health Organization^5^.

*A*. *baumannii* is a successful nosocomial pathogen due to its ability to overcome antibiotic and disinfectant treatments, as well as, to resist desiccation^3,6–10^. A wide variety of nosocomial infections, including pneumonia, bacteremia, meningitis, wound infections, post-surgical infections, (among others), can be caused by this threatening pathogen^11–14^. Previous studies illustrated that when *A*. *baumannii* is exposed to a high-stress environment, such as blood, serum, iron limitation, it can respond and shift the expression of different genes to overcome the imposed stress^15,16^. For example, Jacobs *et al*. showed that a culture of *A*. *baumannii* in the presence of human serum up-regulates the expression of genes coding for iron acquisition, drug efflux pumps, epithelial cell adherence and DNA uptake^15^. Further supporting this observation, human serum albumin (HSA) was shown to trigger the differential expression of a number of key genes involved in the survival and persistence of *A*. *baumannii*^17^. Additionally, HSA-containing fluids (pleural fluid, liquid ascites, human whole blood) caused an increase in the natural transformation frequency of *A*. *baumannii*^18^.

We hypothesized that components (e.g., albumin, proteins, monocytes, granulocytes, and neutralization agents) present in human fluids, such as pleural fluid (PF), alter the response of *A*. *baumannii* such that the bacterium can overcome the stress and survive. Our previous work, which focused on the analysis of genes related to metabolic processes, showed that when *A*. *baumannii* encounters PF a surprisingly large number of genes related to metabolic processes were affected^19^. Due to changes in intracellular pyruvate and phenylalanine metabolism in the presence of PF, we observed that *A*. *baumannii* cytotoxicity and immune evasion were enhanced. This finding illustrates the key role played by bacterial metabolism in the pathophysiology of this bacterium during respiratory infections^19^. In addition, the transcriptomic analysis showed that more than 55% of the genes differentially expressed were associated with metabolic processes, such as glutamate, short chain fatty acid, and styrene metabolism^19^.

Considering the importance of *A*. *baumannii* as one of the leading causes of hospital-acquired pneumonia^20,21^, Wang *et al*. (2014), using the *A*. *baumannii* strain ATCC 17978 and a murine pneumonia model, created a random transposon mutant library and identified that 157 genes were necessary for *A*. *baumannii* to persist in the mouse lung^22^. The genes identified to be necessary for persistence in the pneumonia model, included known virulence factors, efflux pumps, as well as genes involved in metabolic processes, including the transport of amino acid and nucleotides, and stress responses^22^.

The present work builds upon our hypothesis, previous findings, and the importance of *A*. *baumannii* as a respiratory pathogen. In this study, taking advantage of our existing RNA-seq data and with a complementary goal to our previous work^19^, we further explore the impact of PF exposure on *A*. *baumannii* gene expression and on the behavior of traits involved in its pathogenicity.

## Results

### PF modifies stress- and virulence-related gene expression profile in *A.baumannii*

**Figure 1:**
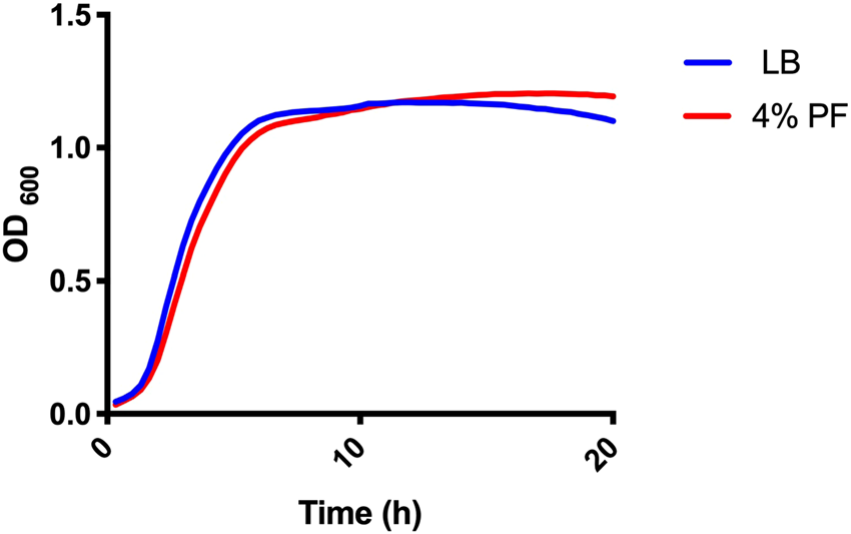
A118 growth curve in LB or LB 4% PF. Strain A118 was grown in LB broth plus or minus 4% PF. Statistical analysis was performed using Mann-Whitney (n = 3).

To determine the effect of PF on *A*. *baumannii*’s pathogenicity traits, we analyzed the available RNA-seq *A*. *baumannii* A118 reads generated from cells incubated in LB with or without the addition of PF^19^. We focused these analyses on genes related to virulence and stress response. Importantly, the growth of A118 is unaffected by the presence of PF in the medium (Fig. 1). Similarly, the growth of other strains, A42 and AB5075, was also not perturbed by PF (Fig. S1); thus, supporting the ability of *A*. *baumannii* to grow in this fluid.

Applying a more stringent analysis, using a fold-change cutoff of log_2_ > 1 (with adjusted *P*-value < 0.05), we obtained a list of 1120 differentially expressed genes (DEGs) associated with a wide variety of functions that may play significant roles in survival within the host. Some these functions included natural transformation, motility, biofilm formation, efflux pumps, two-component systems, type-6 secretion system, fibrinolytic activity, capsule synthesis, hemolytic activity, osmotic stress, DNA-damage responses, and metal resistance as well as antibiotic resistance (Table S1).

### PF alters motility and biofilm formation

**Figure 2:**
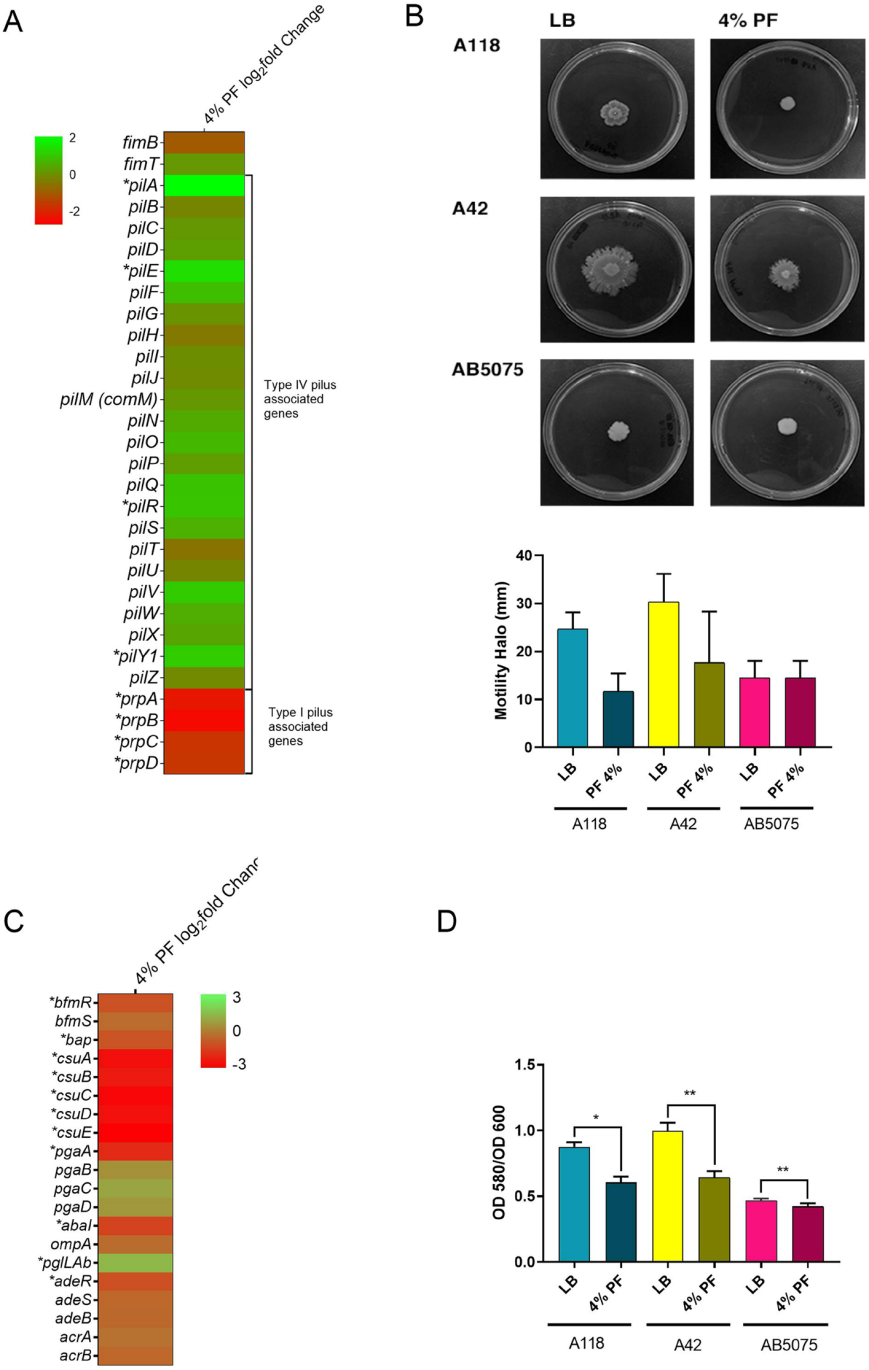
Phenotypic and genetic analysis of surface associated motility genes and biofilm formation. (**A**) Heatmap outlining the differential expression of genes associated with fimbriae biogenesis, structural organization of T4P and motility, and type I pilus. The majority of motility associated genes are up-regulated (green) in the presence of PF. The asterisks represent the DEGs (adjusted *P*-value < 0.05 with log_2_fold change > 1). (**B**) Surface motility assays resulted in a decrease in the diameter of motility (mm) when grown in the presence of PF. Experiments were performed in triplicate, with at least three technical replicates per biological replicate. Statistical analysis (Mann-Whitney test) was performed using GraphPad Prism (GraphPad software, San Diego, CA, USA), and a *P-*value < 0.05 was considered significant. (**C**) A heatmap of the differential expression of genes associated with biofilm formation, including *ompA*, the *csu* operon and its regulator *bfmRS*. Asterisks represent the DEGs (adjusted *P*-value <0.05 with log_2_fold change >1). (**D**) Biofilm assays performed with and without PF show a significant decrease in biofilm formation as represented by OD_580_/OD_600_, when grown in the presence of PF. Experiments were performed in triplicate, with at least three technical replicates per biological replicate. Statistical analysis (Mann-Whitney test) was performed using GraphPad Prism (GraphPad software, San Diego, CA, USA), and a *P-*value < 0.05 was considered significant.

Comparison of mRNA levels between cells cultured in the presence or absence of PF revealed that of 30 genes associated with motility, *pilA*, *pilE*, *pilR*, *pilY1*, and the *prpABCD* operon were DEGs (Table S1). These genes are associated with the type IV pilus (T4P) and type I pilus (Fig. 2A), which play a role in *A*. *baumannii* twitching motility and surface-associated motility, respectively^23,24^. The *prpABCD* operon, together with eight other genes involved in fimbriae biogenesis, motility, and structural organization of T4P were down-regulated (Fig. 2A). To test if these changes in expression levels resulted in phenotypic modifications, we performed surface motility assays in *A*. *baumannii* A118 and two other strains, A42 and AB5075. A reduction by 57.7% and 41.8% in surface motility was observed in strains A118 and A42, respectively (Fig. 2B). In the case of strain AB5075, the presence of PF was correlated with a subtle change in the shape of the spot, but this might be due to marginal bacterial growth rather than motility (Fig. 2B).

The presence of PF in the growth medium also resulted in a reduction in the expression of 16 out of the 20 genes identified associated with biofilm formation (Fig. 2C). Genes belonging to the *csuA/BABCDE* operon, the two-component system (TCS) response regulator *bfmR*, and its sensor kinase *bfmS*^25^, as well as, the ortholog of *bap*, the biofilm-associated protein, were down-regulated (Fig. 2C and See Supplementary Table S1). Supporting this observation, *A*. *baumannii* A118, as well as strains A42, and AB5075, showed a decrease in biofilm formation to different degrees (Fig. 2D). This result is in agreement with our previous observation that biofilm formation by *A*. *baumannii* A118 decreases when cultured in the presence of HSA, which is a component of PF^17^. Both results, taken together suggest that HSA is, at least in part, responsible for the inhibition of biofilm formation caused by PF.

### PF affects antibiotic susceptibility across antibiotic families

**Figure 3:**
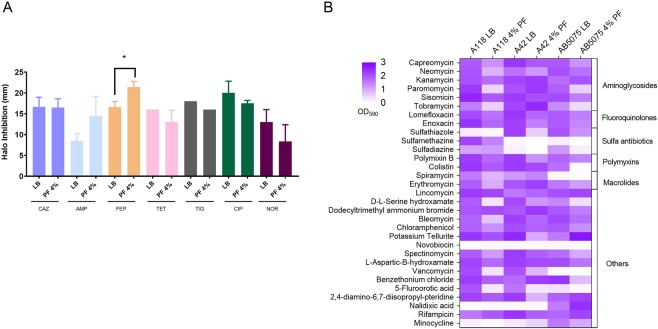
Antimicrobial susceptibility and phenotype microarrays assays. (**A**) Disk-diffusion assays were performed and changes in the halo of inhibition were recorded and an increase in susceptibility was observed for ampicillin, ceftazidime and cefepime in A118 cells cultured with and without PF. AMP: ampicillin, CAZ: ceftazidime, FEP: cefepime, IMP: imipenem, MEM: meropenem, CIP: ciprofloxacin, NOR: norfloxacin, GM: gentamycin, AMK: amikacin, SXT: sulfamethoxazole, TET: tetracycline. (**B**) Heat map of phenotype microarray demonstrating growth of *A*. *baumannii* strains A118, A42 and AB5075, with and without 4% PF in various antibiotics.

We previously observed that that HSA, a component of PF, affected the susceptibility profiles towards β-lactam antibiotics in the A118 strain. To test the effect of PF on antibiotic susceptibility profile of A118, disk diffusion and E-test minimal inhibitory concentration (MIC) measurements were performed^17^. Both methodologies showed an increase in susceptibility towards ampicillin, ceftazidime and cefepime in the presence of PF (Fig. 3A, Supplementary Table S2). On the other hand, no significant changes in susceptibly were observed for the imipenem, meropenem and cefazolin. Phenotype microarray plates further confirmed the susceptibly results for additional β-lactam antibiotics, including cefazolin, ceftriaxone, cephalothin, and carbenicillin, for which a reduction in resistance levels in PF-containing medium was observed (Data not shown). Moreover, in agreement with our previous HSA-induced transcriptomic data^17^, the expression of three A118 *A*. *baumannii* genes associated with resistance to β-lactams, *carO*, *bla*_ADC-99_ and *bla*_OXA-89 (51-like)_, was reduced when PF was present in the culture medium (See Supplementary Table S1). β-Lactam resistance levels were also assessed in two other *A*. *baumannii* strains (A42 and AB5075) that possessed different levels of resistance compared to A118 strain. A42 and AB5075 typically exhibit intermediate and highly levels of drug resistance, respectively^26,27^. Disk diffusion and MIC testing did not reveal any relevant changes in the susceptibility profiles for strains A42 and AB5075 when treated with PF (Supplementary Tables S2 and S3).

The effect of PF on susceptibility profile of the A118 strain to β-lactams led to the question whether there would be an effect on susceptibility levels to other antimicrobials. Accordingly, representatives of different antibiotic families were tested. The presence of PF in the culture medium of *A*. *baumannii* A118 was associated with a significant reduction in susceptibility levels to fluoroquinolones (ciprofloxacin and norfloxacin), tetracycline, tigecycline, and sulfamethoxazole-trimethoprim via disk diffusion (Fig. 3A). This corresponded to a significant reduction in the MIC values for tetracycline, ciprofloxacin, and norfloxacin; less of a reduction was observed with tigecycline (See Supplementary Table S2). The other two strains tested (A42 and AB5075) showed somewhat different behavior. *A*. *baumannii* A42 experienced a decrease in susceptibility to tetracyclines and fluoroquinolones and an increase in resistance for gentamicin (Supplementary Table S3). On the other hand, significant changes were not observed with the highly resistant strain AB5075 (Supplementary Table S3).

To further investigate the effect of PF on susceptibility to different antibiotic agents, phenotype microarrays were also used. This assay, showed that the presence of PF was also associated with various levels of increased susceptibility to aminoglycosides, sulfa-drugs, macrolides, minocycline, chloramphenicol, polymyxins, vancomycin, serine hydroxamate, and fluorotic-acid (Fig. 3B). The PF-mediated enhanced susceptibility to vancomycin was of special interest, because this antibiotic, in combination with colistin and/or doripenem, is effective against extensively drug-resistant (XDR) *A*. *baumannii* strains^28^. Phenotype microarray plates testing A42 and AB5075 revealed that susceptibility towards aminoglycosides, sulfa-drugs, chloramphenicol, polymyxins, vancomycin, serine hydroxamate, or fluorotic-acid was also increased in these strains in the presence of PF (Fig. 3B), as observed for strain A118.

Genes belonging to the three RND-type of efflux pumps that are known to efflux antimicrobials^29,30^ were also affected by PF (See Supplementary Fig. S2A and Table S1). The transcriptomic data revealed that *adeABC and adeIJK* that code for efflux pumps were down-regulated under PF induction (See Supplementary Fig. S2A and Table S1). The genes encoding the two component systems AdeRS and BaeSR involved in efflux pump expression are also down-regulated in the presence of PF (See Supplementary Fig. S2B and Table S1). In contrast, *adeFGH* efflux pump coding genes were up-regulated (See Supplementary Fig. S2A and Table S1). RND-type efflux pumps are known to contribute in resistance to antimicrobials^17,18^, the increase and decrease in the expression of them can explain the antibiotic susceptibility changes observed upon-PF exposure (Fig. 3A,B). The increase in resistance to fluoroquinolones, tetracycline and also tigecycline can be in part explain by the up-regulation of the genes coding for AdeFGH.

### Gene ontology analysis reveals that PF triggers the expression of genes involved in DNA-damage

Molecular pathways involved in *A*. *baumannii’s* adaptive response to PF were identified by gene ontology (GO) enrichment analysis. By interrogating the total list of 1,120 DEGs, we mostly observed low enrichment values for generic, low informative GO terms. Accordingly, we performed further GO enrichment analyses on different subsets of genes filtered by a log_2_fold change differential expression values of 1 or higher (1.58, 2, and 3). The GO term’DNA repair‘ [GO:0006281] showed a statistically significant overrepresentation (adjusted *P*-value < 0.05) by 5.3- and 18.3-fold in DEGs when filtered by a log_2_fold enrichment of 2 and 3, respectively (See Supplementary Fig. S3A). These included genes encoding the proteins RecA, the error-prone DNA polymerase V UmuC, the Holliday junction ATP-dependent DNA helicases RuvA and RuvB, and excinuclease subunits. In addition, the GO term’SOS response‘ [GO:0009432], which includes some of the latter genes, was also enriched in our analysis, showing an even higher overrepresentation by 19.5- and 82-fold in the lists filtered by a log_2_fold enrichment of 2 and 3, respectively (Supplementary Fig. S3A).

To determine whether PF impacts the ability of *A*. *baumannii* to overcome the action of DNA-damaging agents, we first analyzed the effect of mitomycin C (MC), a potent DNA crosslinker that is effective at killing bacteria^31^, on A118, A42, and AB5075 by exposing these strains to 0.2 μg/ml of the antibiotic and measuring cell-kill using colony counts. The three analyzed strains exhibited a 3-fold increase in viability to MC in the presence of PF (See Supplementary Fig. S4A). Also, in plates containing another mutagen, ethidium bromide at 1 mg/ml, only growth of A118 and A42 when treated with PF was observed (Data not shown). In addition, we determined the MICs for levofloxacin and ofloxacin -two antibiotics that target DNA replication- for A118, A42, and AB5075 in the absence and presence of PF. All three strains possessed an increase in fluoroquinolone MIC in the PF treatment condition. As shown in Supplementary Fig. S4B, A. *baumannii* A118 PF-treated bacteria exhibited a 4-fold increase in resistance to levofloxacin and ofloxacin, an extent similar to the PF-mediated increase in expression of the above-mentioned genes involved in DNA repair (Fig. 3A). These results are in concordance with observed disk diffusion and MIC results for the other fluoroquinolones tested (See Supplementary Table S2). Similar results were observed with strains A42 and AB5075 (See Supplementary Fig. S4C,D). Taken together, these results suggest that exposure to PF results in an adaptive response to DNA damage mediated by the expression of DNA-repair genes.

### PF reduces the expression of osmotic stress related genes in *A. baumannii*

**Figure 4:**
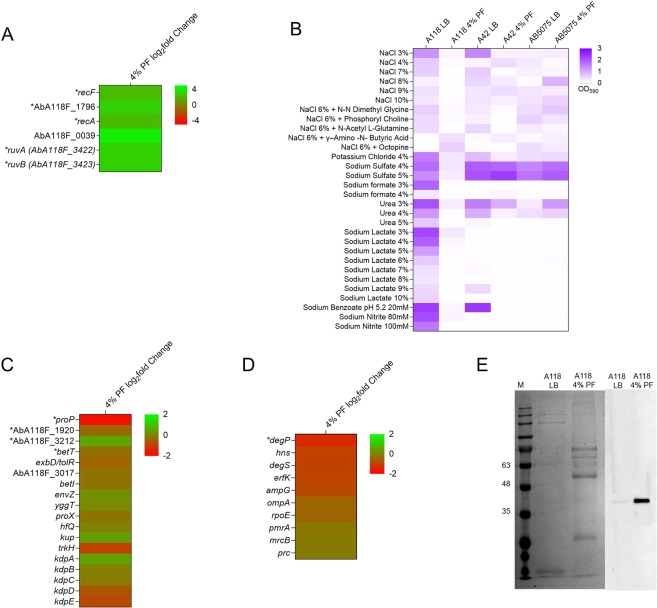
DNA-damage responses, osmotic stress and OMVs associated genes affected by PF. (**A**) Heat map of the differentially expressed genes associated with the DNA-damage response in *A*. *baumannii* strain A118 under 4% PF exposition. (**B**) Phenotype microarray heat map of strain A118 induced with or without PF in various sources of osmolytes. (**C**) Heat map of the differentially expressed genes associated with osmotic stress response. (**D**) Heat map of the differentially expressed genes associated with the OMVs biogenesis in *A*. *baumannii* strain A118 under 4% PF exposition. (**E**) SDS-PAGE (lanes 1 and 2) and Western blot analysis (lanes 3 and 4) of proteins of OMVs from A118. For the western blot analysis, the samples were immunoblotted with a rabbit anti-AbOmpA immune serum. Arrows indicate AbOmpA. Both SDS-PAGE and western blot were cropped to only observe content from strain A118. Brightness and contrast were adjusted to observe less visible bands. The full-length blots/gels are presented in Supplementary Fig. S5.

Changes in the concentration of osmolytes, such as NaCl and KCl, can cause osmotic stress on bacteria^32–34^. The action of PF on the ability of *A*. *baumannii* A118 to cope with various osmotic pressures was determined using phenotype microarrays by screening a broad range of osmolytes. Figure 4B shows that the presence of PF in the growth medium is correlated with a decrease in the tolerance to NaCl, KCl, urea, sodium lactate, sodium benzoate, and sodium nitrite, and others. Genes known to be involved in adaptation to high osmolarity and osmotic stress response were assessed^34^. In concordance with the phenotype microarray results, the expression of most of the genes encoding transporters involved in osmotic response was decreased (Fig. 4C). Also, the genes coding for the TCSs BmfSR, BaeSR, and AdeRS were down-regulated and these TCSs are linked with osmotic stress^10,35^. BaeSR is associated with efflux pump expression, adaptation to changes in environmental osmotic pressure, and it is believed to cross-talk with AdeRS, which is involved in antibiotic resistance and biofilm formation^35,36^. The reduced biofilm formation and decrease viability under high osmolarity can be explained by the down-regulation of *bfmR*. Farrow *et al*. 2018, have shown that BfmR is necessary for the cell to adapt to high salts condition^10^. An increase sensitivity to osmolytes was observed when PF was present, which is further supported by the decreased expression of genes coding for BCC-transporters, K+ transporters, EnvZ, and AdeSR^10,33^. Phenotype microarray assays were also performed on the other two strains, A42 and AB5075 (Fig. 4C). Similarly, to A118, A42 exhibits decreased tolerance to NaCl and urea in the presence of PF, while the presence of PF did not affect the growth in sodium sulfate. For AB5075, less growth was observed in the presence of PF to NaCl, however, in the presence of PF, more growth was observed in sodium sulfate and urea. AB5075 were not able to growth in the presence of sodium lactate and sodium nitrate for both LB and PF conditions, suggesting a low tolerance to this osmolytes. Diverse tolerance to certain osmolytes was observed across the strains, suggesting a strain specific osmotic response.

### PF increases outer membrane vesicles and capsule formation

Since exposure to PF is a stressor, we measured the formation of outer membrane vesicles (OMVs). OMVs are spherical nanovesicles with a diameter of 20–250 nm that form in response to accumulation of misfolded envelope proteins to alleviate cell damage and are also used to secrete virulence factors and tissue-degrading enzymes in *A*. *baumannii*^37,38^. A significant increase in the number of OMVs released was observed when *A*. *baumannii* A118 was exposed to PF (See Supplementary Table S4). Strains A42 and AB5075 also showed an increase in number of OMVs released in the presence of PF. These results strongly suggest that OMV release may be another strategy used by *A*. *baumannii* to respond to the stress caused by the presence of PF (See Supplementary Table S4). Strain A118 possessed the highest increase in OMV release suggesting a higher ability to overcome the stress imposed by PF. This was further supported by a decrease in the expression of genes (e.g., *degP*, *degS, ampG, ompA*) known to be involved in the biogenesis of OMVs (Fig. 4D). Cells with down-regulated levels of periplasmic proteases DegP and DegS or permease AmpG are expected to accumulate misfolded proteins or peptidoglycan fragments in the periplasm, respectively, resulting in an increase in the release of OMVs^39,40^. In addition, previous studies have shown that *A*. *baumannii* ΔAbOmpA mutant increases the release of OMVs^41^.

Previous studies have shown that *A*. *baumannii* can secrete virulence factors in OMVs, such as OmpA and other tissue-degrading enzymes^38^. Using Western blotting and anti-OmpA serum, the cytotoxic OmpA was found in the OMVs of A118 exposed to PF (Fig. 4E). The presence of OmpA in the OMVs of the strains (A42 and AB5075) was also observed (See Supplementary Fig. S5).

Genes involved in capsule formation in *A*. *baumannii* are important for virulence^42–44^. The capsule surrounds the bacterial surface and is essential for serum resistance, optimal growth in human fluids, and resistance to various antimicrobials^45–47^. We found that 11 capsular polysaccharide (K) locus genes in *A*. *baumannii* A118 were up-regulated when PF was added to the growth medium (See Supplementary Fig. S2C and Table S1). As reported by Chin *et al*. (2018), *A*. *baumannii*’s opaque phenotype was associated with the production of a thicker capsule and linked to an increase virulence^48^. The presence of PF did not alter the transition phenotype from opaque to translucent in strain A118 (Data not shown).

## Discussion

Like most bacterial pathogens, *A*. *baumannii* must overcome the hostile conditions it encounters within the human host^1,3,49,50^. Moreover, it must be able to overcome specific and general host defense systems, and survive in contact with human host fluids and tissues^22,25,51–56^. Here, we investigated the effect of PF, a fluid that *A*. *baumannii* must encounter in cases of lung infection, and how *A*. *baumannii* responds to persist and survive. Bacterial stress is expected to increase upon contact with human PF, which includes macrophages, lymphocytes, monocytes, granulocytes, proteins, LDH, and other elements hostile to bacterial infection.

The presence of PF in the *A*. *baumannii* A118 growth medium was correlated with a decrease in motility and biofilm formation together with a reduction in the levels of expression of related structural and regulatory genes^57^. The significant reduction of expression of the *prpABCD* operon, which was identified to be in involved in surface motility^24^, can support the reduced motility in semisolid surface observed in PF-induced cells. Previous studies of clinical isolates of *A*. *baumannii* recovered from different samples (blood and sputum) have shown that depending on the source of the isolate the motility varied^58^. The authors observed that sputum isolates were less motile than blood isolates, suggesting an advantage that can lead to firmly attachment to the alveolar cells^58^. In addition, the reduced expression of the genes involved in biofilm formation, maintenance and maturations (*csuABCDE*, *bap*) concur with the decrease in biofilm under PF treatment. The complex composition of the PF, together, with the wide pathoadaptative response of *A*. *baumannii* cells expose to this fluid, might explain this result.

Although the PF components (albumin, proteins, monocytes, granulocytes, and neutralization agents) responsible for these effects remain to be elucidated, the similarities observed with our previous results using HSA^17^ indicate that this element contributes at least partially to the inhibition of gene expression. The effect of HSA may be more complex however, as it is influenced by other components of the fluids. For example, other authors found that in some *A*. *baumannii* strains the presence of human serum in the growth medium was not associated with changes in biofilm formation^59^. Also, it was previously observed that sputum *A*. *baumannii* isolates, as well as, *A*. *baumannii* ATCC 19606 cells exposure to mucin show robust biofilm formation^58,60^. Further studies using different fluids and a large number of *A*. *baumannii* strains will better clarify the role of HSA and other components of PF on motility and biofilm formation.

Capsules are virulence factors in many bacteria and in *A*. *baumannii*, the K1 capsular polysaccharide was linked to survival in human bodily fluids such as serum^46,47^. Eleven genes within the capsular polysaccharide (K) locus were expressed at higher levels in the presence of PF, a result that was also observed when the growth medium was supplemented with HSA^17^. The *cgmA and ugd* genes, which code for a sulfatase protein and a UDP-glucose dehydrogenase, respectively, were those whose expression was enhanced more prominently.

Response to antibiotics was affected unequally. Susceptibility to some β-lactams increased, possibly due to reduce expression of genes associated with some efflux pumps (*adeABC* and *adeIJK*). Due to the multifactorial and complex nature of antimicrobial resistance mechanisms, the observed down-regulation of β-lactamase genes could not be the only factor attributed or involved in these changes. More studies testing β-lactamase hydrolytic activity under both conditions are required. Whereas, the resistance levels to tetracyclines and fluoroquinolones increased. The increase in levels of expression of *adeFGH* coding genes, capsule formation and increased stress response in the presence of PF could be responsible for this effect. PF also affected expression of TCSs, such as AdeRS, BaeSR, GacSR, BfmSR and PmrAB, but were enhanced in some and depressed in others, suggesting a complex equilibrium in the regulation and expression of genes survival and persistence in changing environments.

Our GO analyses revealed that PF triggers a transcriptional response with an overrepresentation in the expression of genes related with “SOS response” and “DNA damage”. It is important to point out that *Acinetobacter* spp. lack homologs for the SOS-related transcriptional regulator LexA and the cell division repressor SulA^61^. As this bacterial genus does not possess a conventional SOS response system, the PF-mediated differential expression of the above-mentioned genes may be part of the *Acinetobacter* unusual response to DNA damage, probably as a consequence of reactive oxygen species generated by white blood cells present in PF. In particular, the increased expression of RecA could explain our previous observations that the presence of PF was associated with a significant increase in transformation frequencies^18^. RecA, the major enzyme in homologous recombination, participates in numerous cellular processes related to healing damage caused by stress^62^. RecA is also necessary for normal frequency of integration of foreign DNA^63^, a process required in natural transformation. It is not surprising that RecA has been associated with natural competence in many species^64–67^, a state of the cells that could be triggered by DNA damage under stress conditions^65^. In addition, it was shown that RecA is also involved in survival under heat shock and desiccation conditions, as well as, pathogenicity of *A*. *baumannii*. The increase in viability under different DNA-damaging agents supports the RNA-seq data. In addition, GO analysis also revealed overrepresentation of GO terms related with histidine metabolism (See Supplementary Fig. S3A). We found that the entire histidine-utilization (*hut*) operon responsible for catabolism of histidine to glutamate was induced in response to PF (See supplementary Table S1 and Fig. S3B). Additionally, an ortholog of the Zn-binding GTPase ZigA, the Zn uptake repressor (Zur) ortholog and two Zn ABC transporter components also displayed PF-mediated induction of expression in the presence of PF (See supplementary Fig. S3D and Table S1), an overall behavior reminiscent of that observed by Nairn *et al*. (2016) in *A*. *baumannii* ATCC 17978 cells cultured in Zn-limiting conditions^68^. Vertebrate hosts respond to bacterial infections restricting the availability of numerous micronutrients in a process known as nutritional immunity. In some cases, such as those involving iron, the depletion mechanisms have been thoroughly studied. More recently, other metal ions like zinc or manganese were added to the list of restricted micronutrients^69,70^. *A*. *baumannii* is no exception and requires zinc as a structural component of proteins and cofactor of different enzymes. Our results showing an upregulation of genes involved in zinc uptake and histidine catabolism in the presence of PF are most likely due to a response to the presence of high levels of calprotectin (i.e., low levels of available zinc) in the human fluid.

Another known response to stress, the release of OMVs, was also increased in the presence of PF. OMVs contain a range of different components, including virulence factors and nucleic acids, and they have a role in genetic transfer^71^. The increased expression of RecA and OMVs production, observed during treatment with PF, could act together to favor gene transfer. Furthermore, as is the case with T6SS, OMVs play dual roles: competing with other bacteria and increasing virulence. This latter effect is in part due to OmpA, a component of the vesicles known to have a cytotoxic effect^38^. OMVs modulate host immune responses which contribute to the survival and persistence of *A*. *baumannii* in this hostile environment^72^. The role of OMVs in bacterial competition is thought to be due to the suppression of antimicrobial peptides and secretion of toxic proteins^72,73^.

We have previously observed that PF induced an alternative phenylalanine catabolic route utilizing phenylpyruvate, which proved to promote a neutrophil-evasive state^19^. In addition, *A*. *baumannii* amplified its cytotoxicity against murine macrophages and also together with pyruvate, can trigger *A*. *baumannii* virulence that affect human epithelial cell viability^19^. Collectively and complementary, our results illustrate how *A*. *baumannii* can overcome the stress imposed in a hostile environment, not only using bacterial metabolic strategies but also changing the expression of genes associated with pathogenicity and adaptative response to stress to persist and survive. Since a considerable number of genes are affected in the presence of PF, we believe that a multifactorial strategy might be involved in scenarios containing stressors and detrimental conditions that lead to *A*. *baumannii* adaptation and success in extreme conditions.

**Figure 5:**
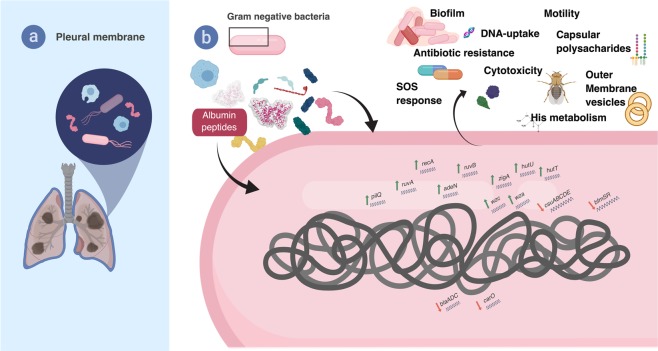
Schematic representation of the model representing *A*. *baumannii* strategies to prevail in adverse environments.

*A*. *baumannii* infections can affect several organs, including the lungs. In these cases, it encounters PF, which likely plays a role in innate host defenses. It is, therefore, an ideal model to understand the interactions between the bacterium and the host, which are reflected in enhancement and reduction in the levels of expression of numerous genes. Our results illustrate these changes and provide a framework to illustrate the impact that human fluids have on the invading *A*. *baumannii* cells, as well as how they adapt to survive and thrive in these hostile conditions (Fig. 5). Future studies comparing changes of gene expression in the presence of other human environments will permit not only the definition of fundamental aspects of different kinds of disease produced by *A*. *baumannii*, but also the design of better therapies.

## Materials and Methods

### Bacterial strains

*A*. *baumannii* strain A118, A42 and AB5075 were used as bacterial models. *A*. *baumannii* strain A118 has been shown to be susceptible to a variety of antibiotics^74,75^, while A42 has shown to have a moderate susceptibility to antimicrobials^27^ and AB5075 is known to be highly virulent and resistant to antimicrobials^26^.

### RNA–seq data analysis

In the present work, previously published RNA-seq reads (GEO accession No GSE131949) corresponding to *A*. *baumannii* A118 incubated with LB or LB 4% PF (Rodman *et al*., 2019) were analyzed as follows. Trimming of low-quality bases at the ends of the reads to a minimum length of 100 bp and removal of Illumina adaptor sequences was performed using Trimmomatic^76^, yielding an average of 16 million paired reads per sample. FastQC (www.bioinformatics.babraham.ac.uk/projects/fastqc/) was used to assess the quality of the reads before and after trimming. Burrows-Wheeler Alignment software (BWA) was used to align the RNA-seq reads to the genome of *Acinetobacter baumannii* A118F, an *A*. *baumannii* A118 reference strain kept at the College of Natural Sciences and Mathematics, California State University Fullerton. *A*. *baumannii* A118F whole genome shotgun project has been deposited at DDBJ/ENA/GenBank under the accession VCCO00000000. The version described in this paper is version VCCO01000000. The alignments were visualized using the Integrated Genome Viewer software^77^. FeatureCounts^78^ was used to calculate the read counts per gene, and differential expression analysis was performed using DEseq.^79^. Features exhibiting FDR <0.05 and log_2_fold change >1 were considered statistically significant.

### Gene ontology analysis

GO terms were retrieved from UniProt for the best BLASTx hits to *A*. *baumannii* A118F genes. Using GO.db Bioconductor annotation data package in R language, GO terms and ancestor terms were assigned for all DEGs from this study. For different subsets of DEGs filtered by log_2_fold change differential expression higher than 1,1.58, 2, or 3, a GO term enrichment analysis was performed for the biological process (BP) category. The enrichment factor was estimated as the ratio between the proportions of genes associated with a particular GO category present in the dataset under analysis, relative to the proportion of the number of genes in this category in the whole genome. p-values were calculated using the Fisher Exact Test and adjusted by the Benjamini-Hochberg method.

### Growth curves

Growth curves were conducted on 96-well plates in triplicate with strains A118, A42 and AB5075 in LB plus or minus 4% PF. Overnight cultures were subcultured 1:50 in LB or LB + 4% PF and incubated for 18 hours at 37 °C with medium shaking. Growth was measured at an OD_600_ every 20 minutes using a Synergy 2 multi-mode plate reader (BioTek, Winooski, VT, USA) and Gen5 microplate reader software (BioTek).

### Motility and biofilm assays

Motility and biofilms assays were performed as previously described^17^. A118 cells were cultured in LB broth with or without 4% PF and incubated with agitation for 18 h at 37 °C. Experiments were performed in triplicate, with at least three technical replicates per biological replicate.

### Opaque and translucent variants

To distinguish between opaque and translucent colonies the previously described protocol was followed^80^. Briefly, A118 cells with and without PF were cultured for 18 h and then plated on agar plates composed of LB broth supplemented with 0.8% agar for 24 h. AB5075 was used as control^48^. The colonies were observed using a stereo microscope with oblique lighting from underneath.^81^

### Susceptibility assays

Mueller-Hinton agar plates were inoculated with 100 µl of a culture of each tested condition (A118 or A118 + PF) after OD adjustment. Antimicrobial commercial disks (BBL, Cockeysville, MD, USA) containing 10 µg of ampicillin (AMP), 30 µg of cefepime (FEP), 30 µg of ceftazidime (CAZ), 10 µg of imipenem (IMP), 10 µg of meropenem (MEM), 5 µg of ciprofloxacin (CIP), 10 µg of norfloxacin, 10 µg of gentamicin (GM), 30 µg of amikacin (AMK), 30 µg of tetracycline (TET), 15 µg of tigecycline (TIG), and 1.25/23.75 µg of trimethoprim-sulfamethoxazole (SXT), were used and the plates were incubated at 37 °C for 18 h. The assays were performed in triplicate. In addition, the minimum inhibitory concentration (MIC) was performed by E-test (Liofilchem, Italy) following CLSI recommendations^82^.

### Phenotype microarray

Bacterial cells were cultured in LB broth with or without 4% PF at 37 °C for 18 hrs. Following the manufacturer instructions (Biolog, CA, USA), inoculation and preparation was done and PM9, PM10, PM11C and PM12B microplates were used. Plates were incubated under aerobic conditions for 24 h at 37 °C and results were read at OD_590_ using the multimodal plate reader SpectraMax3.

### Vesicles formation

Outer membrane vesicles were prepared as follows. Overnight cultures of *A*. *baumannii* A118 in LB broth with or without 4% PF were grown for 18 h at 37 °C at 200 rpm. The culture was centrifuged, and supernatant was collected and filtered with a 0.45 µm filter (Genesee Scientific, San Diego, CA). The supernatant fraction was precipitated with 55% ammonium sulfate, incubated for 18 h at 4 °C with stirring. The resulting fraction was centrifuged, and the pellet was resuspended in 1XPBS. The resuspended pellet was then dialyzed with 12–14 kDa Spectra/Por® 2 Standard RC Dry Dialysis Tubing (Waltham, MA, USA) using 100 volumes of 1X PBS overnight at 4 °C. The dialyzed sample was filtered with a 0.45 µm filter and ultracentrifuged with a bed of 40% sucrose at 150,000 × g with a Ti90/2773 rotor. The pellet (OMVs) were resuspended and the OMV’s were quantified using Pierce™ BCA Protein Assay Kit Thermo Scientific (Waltham, MA, USA). Lipids were quantified using the Cell Biolabs Lipid Quantification Kit (San Diego, CA). Subsequently, proteins were fractionated by 12% polyacrylamide gels and then sodium dodecyl sulfate-polyacrylamide gel electrophoresis (SDS-PAGE). was performed. Proteins were then electrotransferred onto a nitrocellulose membrane. The membrane was probed with anti-OmpA serum, and immunocomplexes were detected via chemiluminescence using protein A labeled with horseradish peroxidase.

### Statistical analysis

Experiments were performed at least in technical and biological triplicate and statistical analysis (Mann-Whitney test) was performed using GraphPad Prism (GraphPad software, San Diego, CA, USA). A *P-*value < 0.05 was considered significant.

All procedures performed in this study were in accordance with the CSUF Institutional Biosafety Committee Approval plan (DBH117-01) and are in compliance with the NIH, CDC, OSHA and other environmental and occupational regulations.

## Supplementary information

Supplementary material

Table Supplementary 1 (S1)
