# Supplementary material for: Human pleural fluid triggers global changes in the transcriptional landscape of Acinetobacter baumannii as an adaptive response to stress
Source: Sci Rep. 2019 Nov 21;9:17251. doi: 10.1038/s41598-019-53847-2 (PMC6872806; doi:10.1038/s41598-019-53847-2)
Supplement: Supplementary file 1 — Supplementary material [file 41598_2019_53847_MOESM1_ESM.pdf]

Human pleural fluid triggers global changes in the transcriptional landscape of *Acinetobacter baumannii* as an adaptive response to stress

Jasmine Martinez[1], Jennifer S. Fernandez[1], Christine Liu[1], Amparo Hoard[1], Anthony Mendoza[1], Jun Nakanouchi[1], Nyah Rodman[1], Robert Courville[1], Marisel R. Tuttobene[1], Carolina Lopez[2], Lisandro J. Gonzalez[2], Parvin Shahrestani[1], Krisztina Papp-Wallace[3,4,5], Alejandro J. Vila[2], Marcelo E. Tolmasky[1], Robert A. Bonomo[3,4,5], Rodrigo Sieira[6], Maria Soledad Ramirez[1*].

[1]Center for Applied Biotechnology Studies, Department of Biological Science, College of Natural Sciences and Mathematics, California State University Fullerton, Fullerton, California, USA, [2]Instituto de Biología Molecular y Celular de Rosario (IBR, CONICET-UNR), Rosario, Argentina, [3]Medical Service and GRECC, Louis Stokes Cleveland Department of Veterans Affairs Medical Center, Cleveland, Ohio, USA, [4]Departments of Medicine, Pharmacology, Molecular Biology and Microbiology, Biochemistry, Proteomics and Bioinformatics, Case Western Reserve University School of Medicine, Cleveland, Ohio, USA, [5]CWRU-Cleveland VAMC Center for Antimicrobial Resistance and Epidemiology (Case VA CARES), Cleveland, Ohio, USA, [6]Fundación Instituto Leloir – IIBBA CONICET, Buenos Aires, Argentina.

Running Title: PF changes *A. baumannii* behavior

Keywords: *Acinetobacter baumannii*, human pleural fluid, serum albumin, antibiotic resistance, biofilm, persistence, survival, metabolism, transcriptomic analysis.

***Corresponding author.**
Mailing address
María Soledad Ramírez, PhD.
Assistant Professor
Dept. Biological Science
California State University Fullerton
800 N State College Blvd
Fullerton, CA 92831
e-mail: msramirez@fullerton.edu/ Tel: +1 657-278-4562

SUPPLEMENTARY MATERIAL

Supplementary Fig 1. A42 and AB5075 growth curves in LB or LB 4% PF. Strains A) A42 and B) AB5075 were grown in LB broth plus or minus 4% PF. Growth curves were conducted in triplicate. Statistical analysis was performed using Mann-Whitney (n=3).

Supplementary Fig 2. Differential expression of genes related to the A) the RND-Type efflux pumps, B) two-component systems, and C) capsular polysaccharide (K) locus, in *A. baumannii* strain A118 under exposure to PF. Genes with an asterisk represent a *P*-value of < 0.05. Asterisk indicates an adjusted *P*-value < 0.05 and log_2fold change > 1.

Supplementary Fig. 3. Analysis of PF-treated *A. baumannii* A118 RNA-seq data. A) GO term enrichment (Benjamini-Hochberg adjusted *P*-value < 0.05) of four terms of the category 'Biological process' in different subsets of DEGs filtered by log_2fold change higher than 1, 1.58, 2 or 3. B-D) Representative images of the alignment of RNA-seq reads of LB-treated (orange) or LB 4% PF-treated (blue) *A. baumannii* A118 at genomic regions encompassing genes related with (B histidine catabolism, C) zinc homeostasis regulation and uptake or D) the Zn-binding GTPase ZigA.

Supplementary Fig 4. DNA-damanging agents' effect on *A. baumannii* viability. A) Mitomycin C (MC) survival of A118, A42 and AB5075. The cells were gown in LB broth plus or minus 4% PF and then serially diluted in agar plates containing MC 0.2 ug/ml. Strains B) A118, C) A42 and D) AB5075 grew in LB broth plus or minus 4% PF were used to performed ofloxacin (OFX) and levofloxacin (LEV) susceptibility. minimum inhibitory concentration (MIC) was performed by E-test (Liofilchem, Italy) following CLSI recommendations [87].

Supplementary Fig 5. Original SDS page and western blot of proteins expressed in OMV's of strain A118.

Supplementary Table S1. Differential gene expression analysis. RNA-seq read counts of four biological replicates of LB- or LB 4% PF-treated *A. baumannii* A118 were analyzed using the DEseq software. For each hit, gene ID, average base mean, base mean group A (LB-treated), base mean group B (LB 4% PF-treated), fold-change, log_2fold change, *p*-value, Benjamini-Hochberg adjusted *P*-value, and gene description/function are provided (see excel file).

Supplementary Table S2. Minimal Inhibitory Concentrations of *A. baumannii* strains A118, A42, AB5075 with or without exposure to 4% PF.

Supplementary Table S3. Disk diffusion assays of *A. baumannii* strains A42, and AB5075 under exposure with or without 4% PF.

Supplementary Table S4. BCA and Lipid quantification of extracted OMVs of *A. baumannii* strains A118, A42, and AB5075 with or without 4% PF.

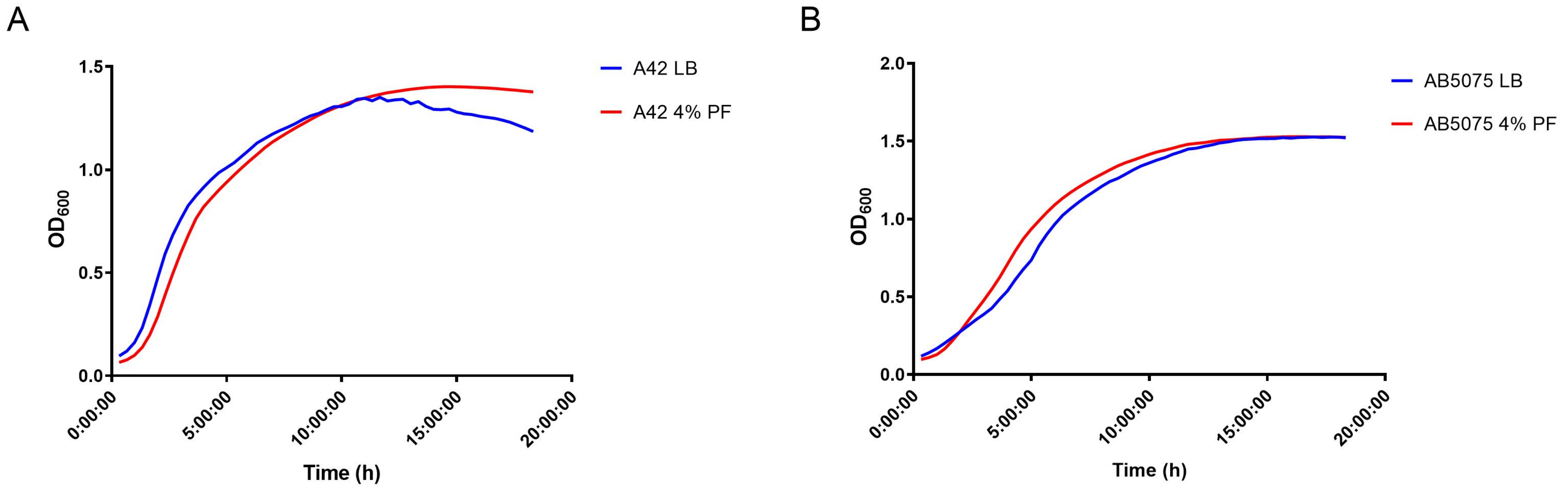
A
1.5
1.0
0.5
0.0
OD600
0:00:00
5:00:00
10:00:00
15:00:00
20:00:00
Time (h)
A42 LB
A42 4% PF
B
2.0
1.5
1.0
0.5
0.0
OD600
0:00:00
5:00:00
10:00:00
15:00:00
20:00:00
Time (h)
AB5075 LB
AB5075 4% PF

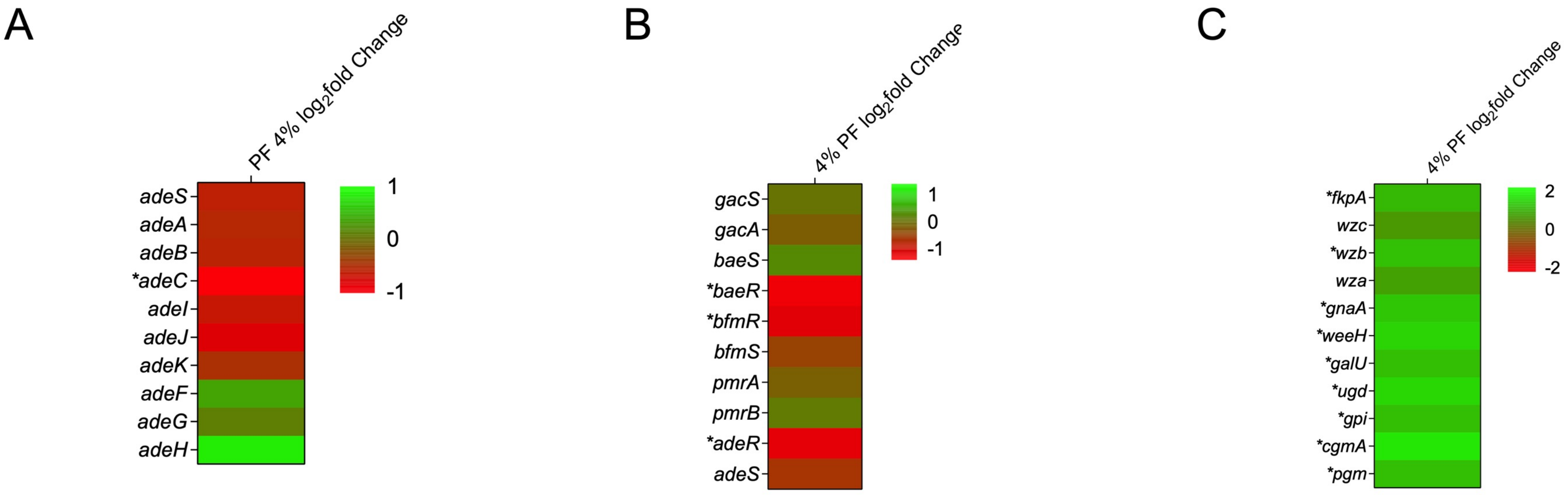
A
PF 4% log2fold Change
adeS
adeA
adeB
*adeC
adeI
adeJ
adeK
adeF
adeG
adeH
1
0
-1
B
4% PF log2fold Change
gacS
gacA
baeS
*baeR
*bfmR
bfmS
pmrA
pmrB
*adeR
adeS
1
0
-1
C
4% PF log2fold Change
*fkpA
wzc
*wzb
wza
*gnaA
*weeH
*galU
*ugd
*gpi
*cgmA
*pgm
2
0
-2

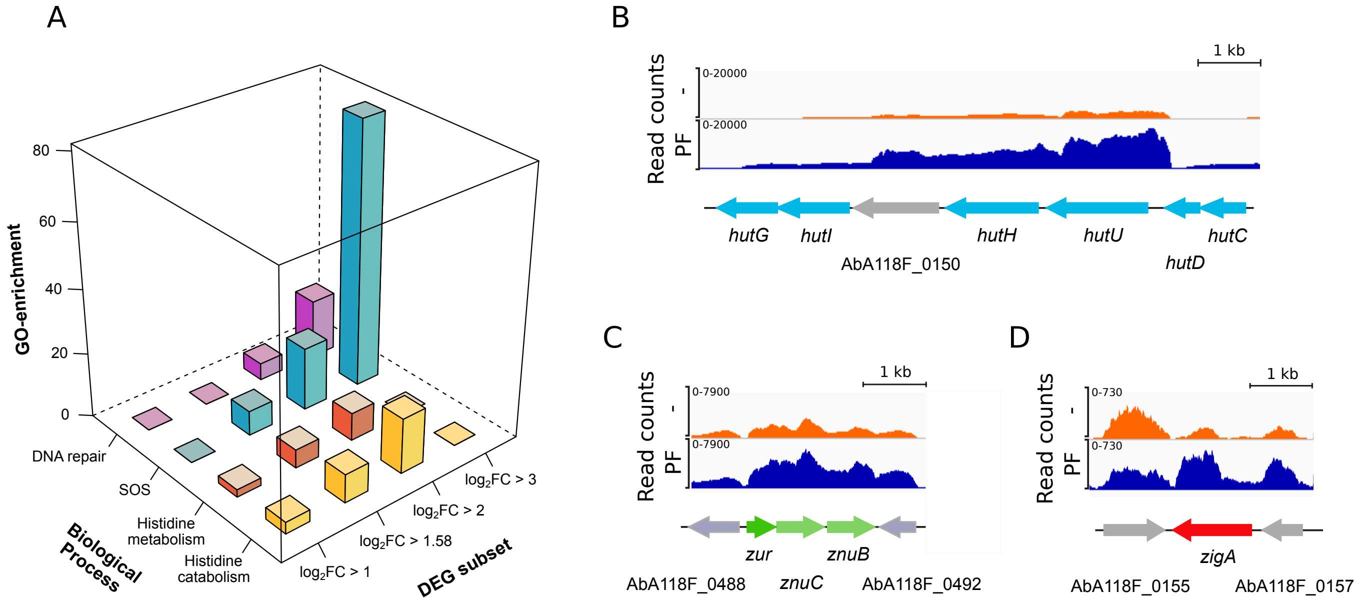

A
GO-enrichment
80
60
40
20
0
DNA repair
SOS
Histidine metabolism
Histidine catabolism
Biological Process
log2FC > 1
log2FC > 1.58
log2FC > 2
log2FC > 3
DEG subset
B
1 kb
Read counts
-
PF
0-20000
0-20000
hutG
hutI
AbA118F_0150
hutH
hutU
hutD
hutC
C
1 kb
Read counts
-
PF
0-7900
0-7900
zur
znuC
znuB
AbA118F_0488
AbA118F_0492
D
1 kb
Read counts
-
PF
0-730
0-730
zigA
AbA118F_0155
AbA118F_0157

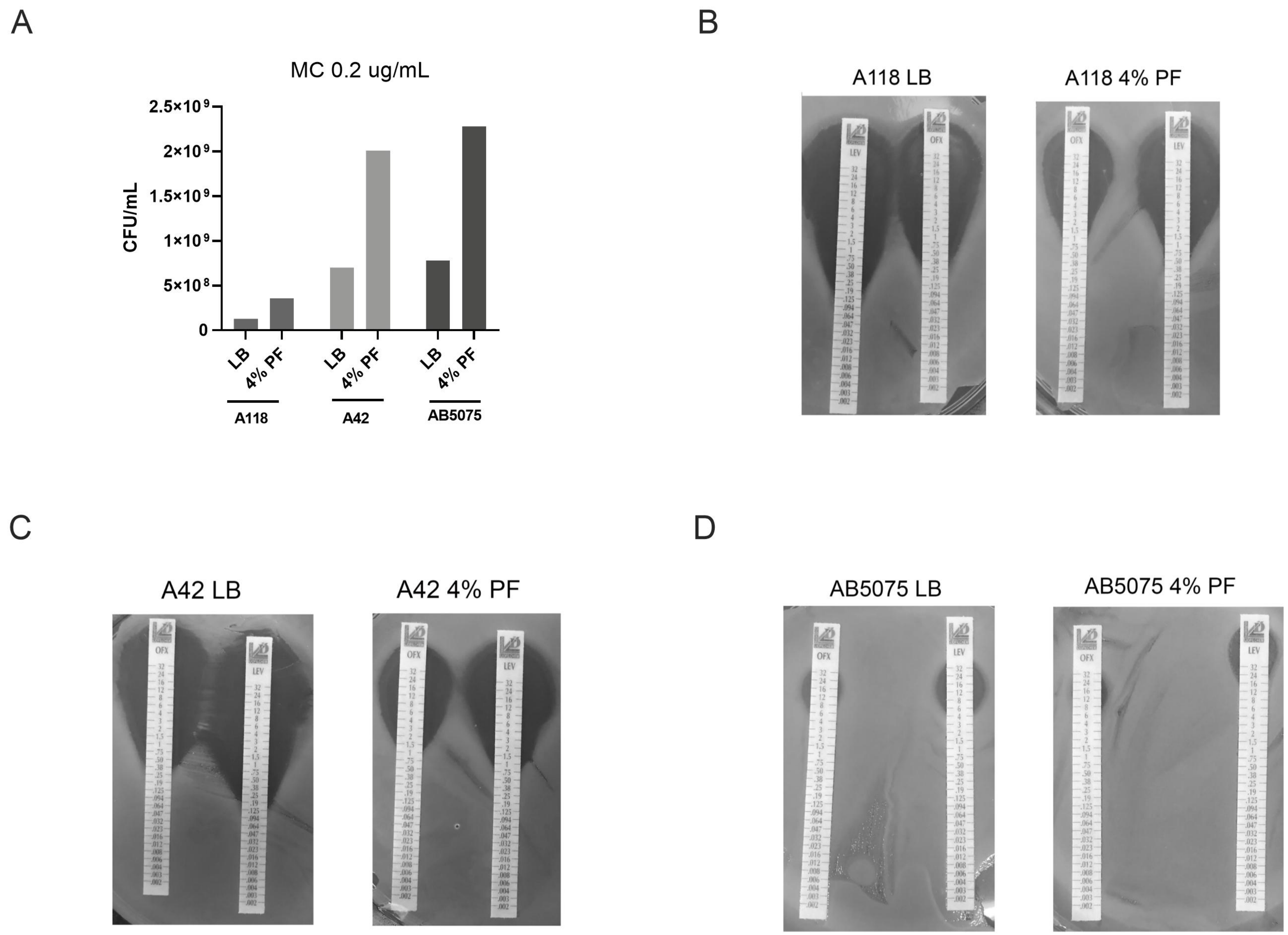
A
MC 0.2 ug/mL
CFU/mL
2.5×10⁹
2×10⁹
1.5×10⁹
1×10⁹
5×10⁸
0
LB
4% PF
A118
A42
AB5075
B
A118 LB
A118 4% PF
C
A42 LB
A42 4% PF
D
AB5075 LB
AB5075 4% PF

A

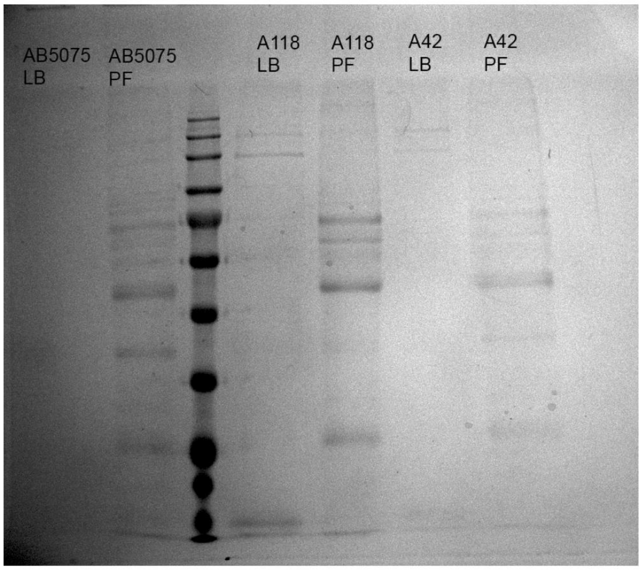

AB5075 LB
AB5075 PF
A118 LB
A118 PF
A42 LB
A42 PF

B

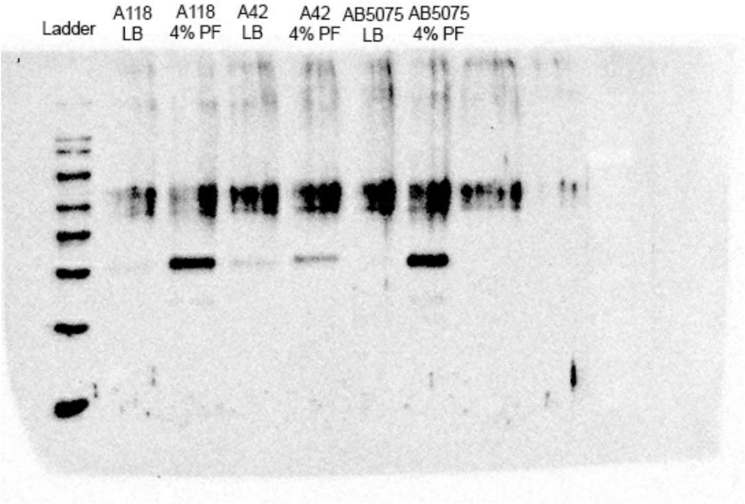

Ladder
A118 LB
A118 4% PF
A42 LB
A42 4% PF
AB5075 LB
AB5075 4% PF

Table S2. Minimal Inhibitory Concentrations of A. baumannii strains A118, A42, AB5075 with or without exposure to 4% PF.

	Minimal Inhibitory Concentration ug/mL				
Strains	MEM	IMP	CAZ	NOR	CIP
A118 LB	0.38	0.38	4	3	0.125
A118 PF 4%	0.75	0.28	4	12	1
A42 LB	0.38	0.38	4	3	0.19
A42 PF 4%	0.65	0.29	12	16	1
AB5075 LB	>32	>32	>256	ND	ND
AB5075 PF 4%	>32	>32	>256	ND	ND

Table S3. Disk diffusion assays of A. baumannii strains A42, and AB5075 under exposure with or without 4% PF.

	Mean Halo of Inhibition (mm)											
Strain	AMP	CAZ	FEP	IMP	MEM	CIP	NOR	GM	AMK	SXT	TET	TIG
A42 LB	11	15	15	30	23	20	14	17	18	14	17	16
A42 PF 4%	7	11	13	31	22	15	11	11	20	17	14	15
AB5075 LB	7	6	6	11	8	6	6	7	9.5	6	14	16
AB5075 PF 4%	7	6	6	10	8	6	6	8	10	6	13.5	16

AMP: Ampicillin, CAZ: Ceftazidime, FEP: Cefepime, IMP: Imipenem, MEM: Meropenem, CIP: Ciprofloxacin, NOR: Norfloxacin, GM: Gentamycin, AMK: Amikacin, SXT: Sulfamethoxazole, TET: Tetracycline.

Table S4. BCA and Lipid quantification of extracted OMVs of A. baumannii strains A118, A42, and AB5075 with or without 4% PF

Strains	BCA ug/mL	Lipid ug/mL
A118 LB	0.34	212.07
A118 4% PF	0.64	644.00
A42 LB	0.07	10.30
A42 4% PF	0.27	396.17
AB5075 LB	0.00	0.00
AB5075 4% PF	0.19	354.14
